# Bubble-Mediated Large-Scale Hierarchical Assembly of Ultrathin Pt Nanowire Network Monolayer at Gas/Liquid Interfaces

**DOI:** 10.1021/acsnano.3c04771

**Published:** 2023-07-06

**Authors:** Enbo Zhu, Yang Liu, Jin Huang, Ao Zhang, Bosi Peng, Zeyan Liu, Haotian Liu, Jiaji Yu, Yan-Ruide Li, Lili Yang, Xiangfeng Duan, Yu Huang

**Affiliations:** ^†^Department of Materials Science and Engineering, ^Δ^Department of Chemistry and Biochemistry, ^‡^Department of Microbiology, Immunology & Molecular Genetics, ^∥^California NanoSystems Institute, University of California, Los Angeles, California 90095, United States

**Keywords:** nanowire network sheet, hierarchical assembly, surface tension, peptide, electrocatalyst

## Abstract

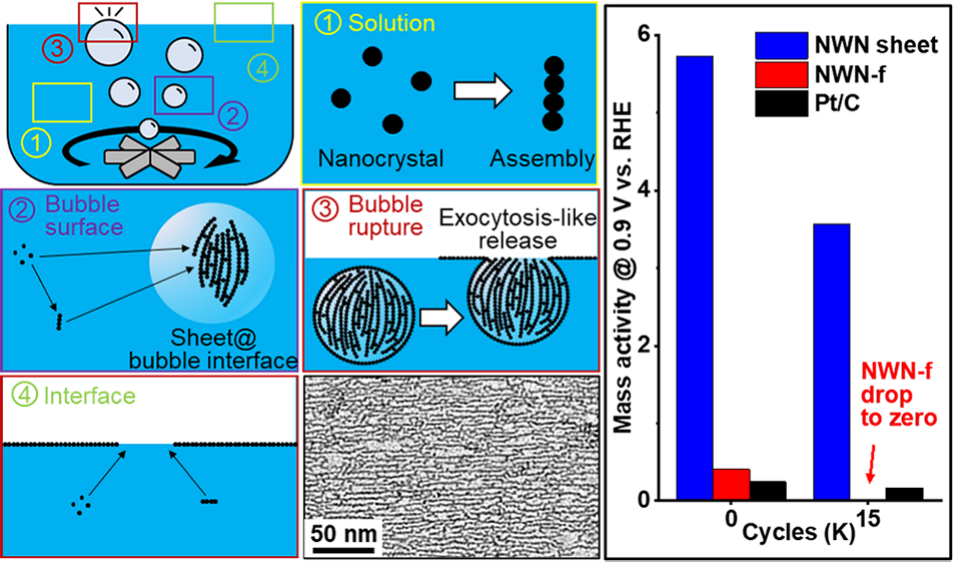

Extensive macroscale two-dimensional (2-D) platinum (Pt) nanowire network (NWN) sheets are created through a hierarchical self-assembly process with the aid of biomolecular ligands. The Pt NWN sheet is assembled from the attachment growth of 1.9 nm-sized 0-D nanocrystals into 1-D nanowires featuring a high density of grain boundaries, which then interconnect to form monolayer network structures extending into centimeter-scale size. Further investigation into the formation mechanism reveals that the initial emergence of NWN sheets occurs at the gas/liquid interfaces of the bubbles produced by sodium borohydride (NaBH_4_) during the synthesis process. Upon the rupture of these bubbles, an exocytosis-like process releases the Pt NWN sheets at the gas/liquid surface, which subsequently merge into a continuous monolayer Pt NWN sheet. The Pt NWN sheets exhibit outstanding oxygen reduction reaction (ORR) activities, with specific and mass activities 12.0 times and 21.2 times greater, respectively, than those of current state-of-the-art commercial Pt/C electrocatalysts.

Morphology and shape anisotropy are critical factors influencing material properties, requiring precise control in the field of chemical sciences.^1−8^ The synthesis of one-dimensional (1-D) and 2-D anisotropic nanostructures has attracted considerable attention in fundamental research, revealing desired properties and functionalities for a variety of applications.^9−18^ The synthesis of an anisotropic nanostructure could be achieved through crystal growth originating from a seed.^19^ Nevertheless, for metal nanoparticles, due to the high symmetry associated with the cubic lattice adopted by the majority of metals, inducing anisotropic growth from seed during colloidal synthesis remains a challenge.^20^ An alternative approach for synthesizing these anisotropic nanostructures is through the assembly of nanoscale building blocks,^21^ where biomolecules may serve as effective ligands due to their highly specific surface recognition properties.^10,22^ By assembling nanometer-scale building blocks, large-scale structures on the order of micrometers and beyond can be created,^23−27^ displaying pronounced anisotropy. Meanwhile, the performance of nanocatalysts is closely linked to their morphology^28−31^ and surface structures,^28−30,32−34^ both of which are heavily influenced by the anisotropy.^35−37^ Current commercial electrocatalysts rely mostly on 0-D nanocrystals, but studies show that anisotropic 1-D and 2-D nanostructures can enhance catalytic activity due to structural anisotropy and improved surface utilization,^35,38−41^ where these highly anisotropic assembled nanostructures can find their contributions.

## Results and Discussion

### Synthesis and Characterization of the Platinum (Pt) Nanowire Network (NWN) Sheet

**Figure 1:**
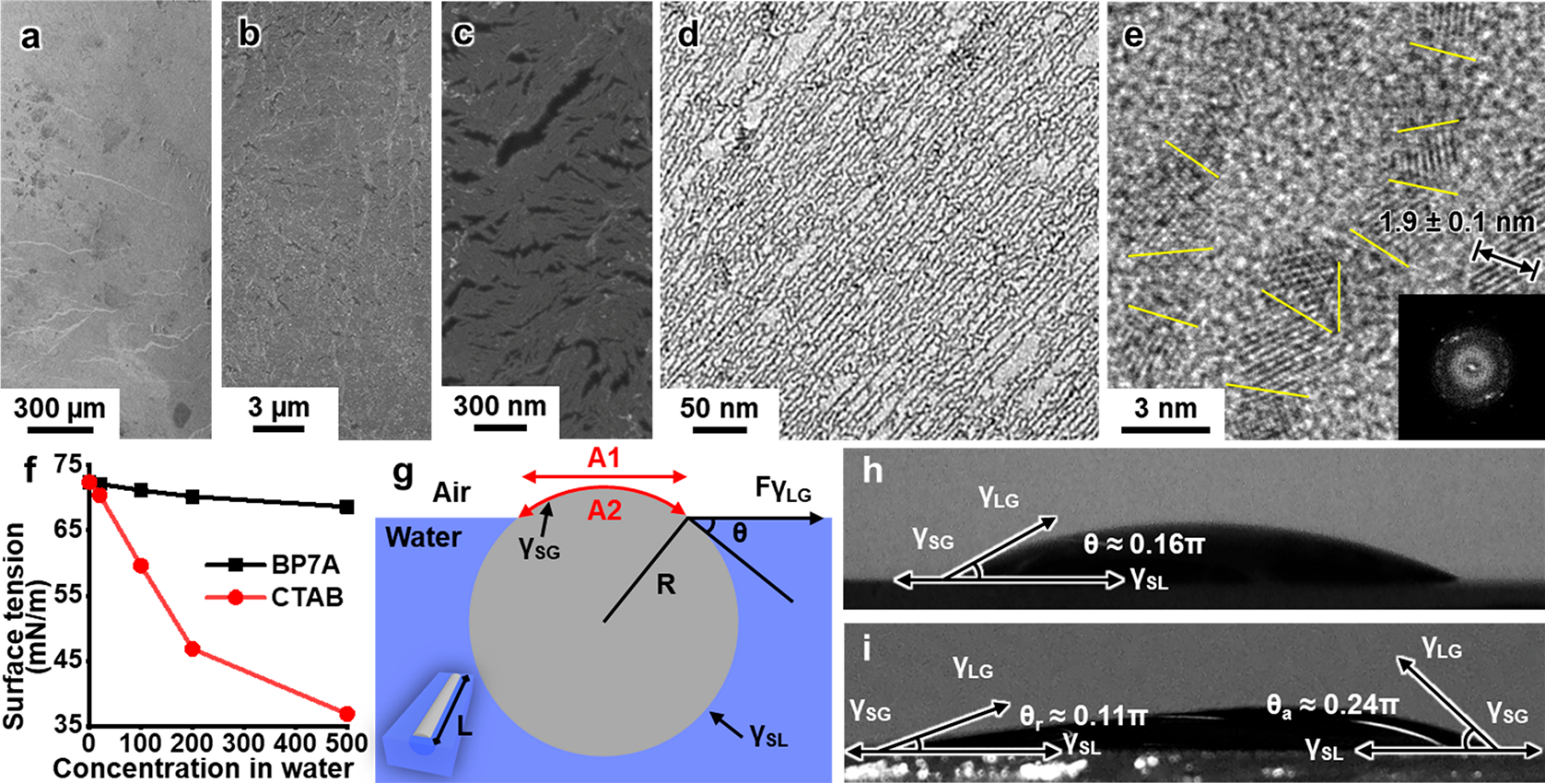
Surface tension stabilization of a monolayer Pt nanowire network (NWN) sheet at the gas/liquid interface. (a–c) Representative SEM images show the monolayer NWN sheet under different magnifications. (d) A representative TEM image reveals the nanowires comprising the monolayer network. (e). A representative HRTEM image shows the grain boundaries marked with yellow lines, with the inset showing the fast Fourier transform (FFT) of the image. (f) Surface tension of water with different concentrations of BP7A and CTAB at 20 °C. (g) Schematic representation of a nanowire pinned at the gas/liquid interface. (h) Static contact angle measurement and (i) dynamic contact angle measurement of a 120 μg/mL peptide BP7A solution on a Pt-NWN-sheet-coated silica wafer.

In this study, we present a facile and ecofriendly method for synthesizing ultrathin sheets of Pt NWNs using a bioinspired surfactant, the BP7A peptide (Ac-TLHVSSY-CONH_2_). The BP7A peptide was identified through the phage display technique as an effective binding agent for multigrain Pt wire surfaces.^36,42^ A typical synthesis was carried out at room temperature (20 °C) in an aqueous solution containing 120 μg/mL of BP7A. (See details in the Experimental Methods section.) A precursor of 1 mM chloroplatinic acid hydrate (H_2_PtCl_6_) was mixed with the solution, followed by the addition of 5 mM ascorbic acid (C_6_H_8_O_6_) and 1.6 mM sodium borohydride (NaBH_4_) as the reducing agents. A magnetic stir bar was employed with an initial stirring rate of 1500 rpm on the injection of NaBH_4_ to ensure fast sufficient mixing. After 5 s, the stirring rate was reduced to 300 rpm. After 30 min, an extended sheet (up to centimeter scale) of nanowires was observed floating at the surface of the solution (Figure S1). The sheets were then transferred onto a silica wafer or a carbon-film grid for characterization by scanning electron microscopy (SEM) and transmission electron microscopy (TEM), respectively. The combination of both imaging techniques revealed that the extended sheets were comprised of uniform monolayer of nanowire network structures (Figure 1a–d), which was hence termed a NWN sheet (Figure 1a–d). High-resolution TEM (HRTEM) show that Pt NWN comprised small Pt nanograins with an average size of ∼1.9 ± 0.1 nm (from 50 random sites examined) with abundant grain boundaries (Figure 1e), consistent with previous studies.^36,43^ The nanowires formed networks that extended from nanometers to micrometers and all the way to centimeter scale (Figure S1). The images also reveal that the NWN sheet is composed of only a monolayer of Pt NNW, whose thickness is at the same scale of the size of the Pt nanograins, leading to an extra high aspect ratio (sheet length/sheet thickness) exceeding 10^7^, highlighting the high anisotropy of the synthesized structure.

It was found that peptide BP7A played an important role in the self-assembly process. BP7A was previously selected to display specific binding affinity to grain boundaries of Pt crystals and subsequently used to promote the attachment growth from 0-D Pt nanocrystals into 1-D Pt nanowires (Figure S2a).^36,37,43^ During the synthesis and assembly process of the ultrathin Pt NWN sheets, we found that the concentration of BP7A played a crucial role in the morphology of the formed nanowires. When a proper concentration of BP7A (120 μg/mL) was used, most of the 0-D nanocrystals selectively attach head to toe to form 1-D Pt nanowires, with a portion of the nanocrystals attached to the sides of the existing nanowires forming branches and connecting nanowires into a network structure (Figure S2b). When the BP7A concentration was reduced to 20 μg/mL, insufficient binding on the nanocrystals can lead to formation of multipod clusters and agglomeration. (Figure S2c). Conversely, when the BP7A concentration was increased to 400 μg/mL, excessive binding of the peptide to the ends of the nanowires obstructed the available sites for Pt nanocrystal attachment, resulting in the formation of short, disconnected nanowires instead of an interconnected, extensive nanowire network (Figure S2d).

### Surface Tension Pins NWN at the Gas/Liquid Interface

This 2-D monolayer NWN sheet structure stood out due to its confinement at the gas/liquid interface, where we believe the surface tension played an important role. Water, a solvent with one of the highest surface tensions, was used in the Pt NWN sheet synthesis, which contributed to the high surface tension. This was notably different from other commonly used solvents such as *N*,*N*-dimethylformamide (DMF), which exhibited lower surface tensions (Figure S3). Additionally, the synthesis of the Pt NWN sheet was carried out at room temperature and atmospheric pressure. This approach is in stark contrast to conventional hydrothermal and solvothermal methods, which necessitate elevated temperature and pressure conditions, consequently leading to a substantial reduction in solvent surface tension.^44^ More importantly, the BP7A peptide employed in the synthesis exerts minimal influence on the solvent surface tension, even at concentrations up to 500 μg/mL. This observation contrasts with traditional surfactants, such as hexadecyltrimethylammonium bromide (CTAB), which are known to significantly reduce the surface tension of solvents (Figure 1f). The preserved high surface tension at the water/gas interface then led to the pinning and preservation of the Pt NWN at the interface (Figure 1g). This distinguishing characteristic sets the Pt NWN sheet synthesis apart from other methods.

The role of surface tension in pinning and maintaining the Pt NWN structure at the water/gas interface was further analyzed. The surface tension of a BP7A solution (120 μg/mL) was measured as γ_*LG*_ = 68.64 mN/m by the Wilhelmy plate method. (See details in the Experimental Methods section.) The ultrathin feature of the nanowires (diameter *D* ≈ 1.9 nm) resulted in a very small Eötvös number:
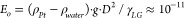
where ρ_*Pt*_ = 21.4 g.cm^–3^ denotes the density of Pt, ρ_*water*_ = 1 g.cm^–3^ denotes the density of water, and *g* = 9.8 m.s^–2^ is the gravitational acceleration. Because *E*_*o*_ ≪ 1, the gravity and buoyancy forces can be ignored in comparison to the surface tension. To account for thermodynamic considerations, the energy reduction from surface tension (*ΔG*) was compared to the thermal motion energy (*kT*) for a nanowire with radius *R*, length *L*, contact angle θ, and surface tensions γ_*SG*_ (solid/gas), γ_*LG*_ (liquid/gas), and γ_*SL*_ (solid/liquid), as illustrated in Figure 1g. The energy reduction by surface tension is given by
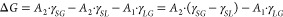
which, after combining with Young’s equation,^45^ γ_*SG*_*= γ*_*SL*_ + γ_*LG*_·cos θ, becomes
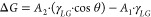


Upon combining with the geometry relations, *A*_1_ = *L*·(2*R*·sin θ) and *A*_2_ = *L*·*R*·(2θ), we get
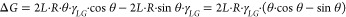
where *R* = 0.95 nm and γ_*LG*_ = 68.64 mN/m. The static contact angle between the solution and Pt NWN sheet coated SiO_2_/Si wafer (see details in the Experimental Methods section) was measured as θ = 0.16π (Figure 1h). Therefore
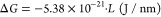
At room temperature (20 °C), *kT* = 4.04 × 10^–21^ J. Δ*G* becomes <−10*kT*, when *L* > 7.51 nm. This indicates that after assembling the first four nanocrystals (each ca. 1.9 nm), the surface tension can stabilize the nanowire on the gas/liquid interface over the Brownian motion. The stabilization or pinning of the Pt NWN at the liquid/gas interface increases with growing nanowire length; e.g., a nanowire consisting of 40 nanocrystals reaches *L* > 75.09 nm and Δ*G* < −100*kT*, and the Pt NWN becomes irreversibly pinned at the interface.^46^ Moreover, to eliminate the effect of time, evaporations, and local inhomogeneities on the surface tension, dynamic contact angles were measured (Figure 1i), where the advancing angle θ_*a*_ = 0.24π, and the receding angle θ_*r*_ = 0.11π provided the upper and lower limits of the true contact angle. According to the dynamic contact angle measurements, the minimal assembly length to achieve stable interface pinning (Δ*G* < −10*kT*) is 2.3 nm −22.82 nm, and the minimal assembly length to reach irreversible interface pinning (Δ*G* < −100*kT*) is 23.0–228.2 nm. (See details in the Experimental Methods section.)

### Role of Bubbles in the Formation of Pt Monolayer NWN Sheets

**Figure 2:**
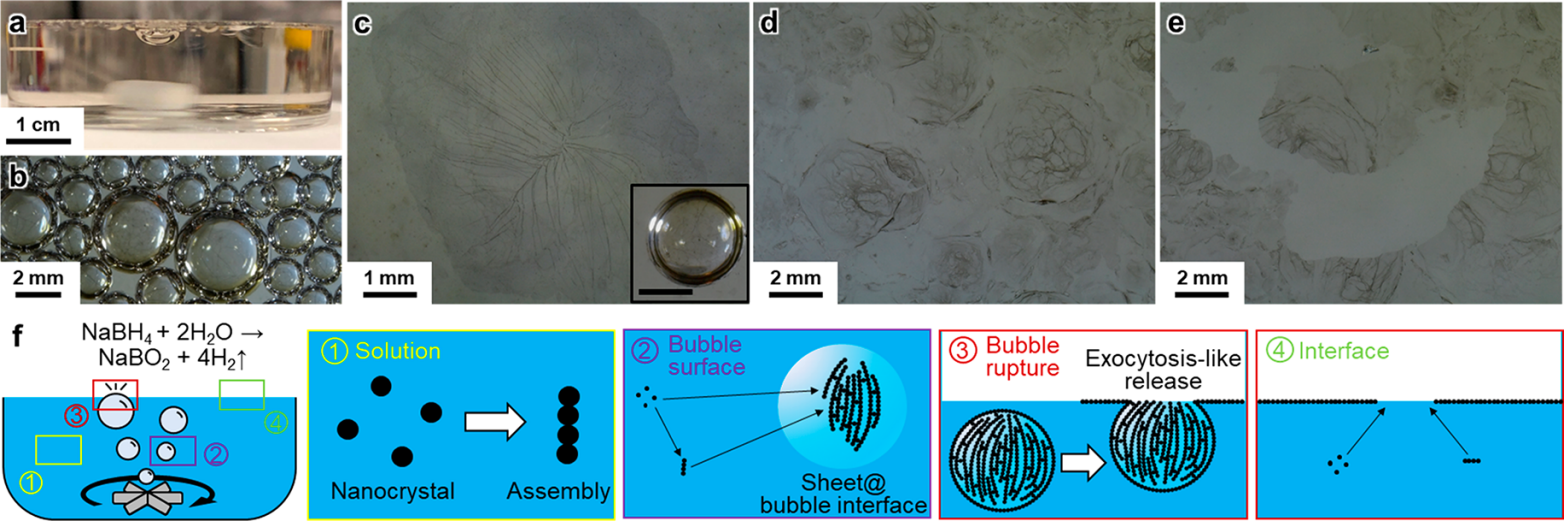
The NWN sheet formation mediated by bubbles. (a) Bubbles generated during the synthesis process. (b) NWN sheets developed at the gas/liquid interfaces of bubbles. (c) An NWN sheet developed in a single bubble was released after the bubble ruptured on the liquid surface. The inset is the bubble before rupturing with a 1 mm scale bar. (d) Vestiges of the NWN sheets remained in the final fused NWN sheet, originating from the bubbles. (e) Artificially breaking the final NWN sheet in (d) showed neat fractures not related to the circular vestiges, indicating that the final NWN sheet was a whole piece. (f) Schematics showing how the monolayer NWN sheet formed at the bubble interfaces and then released to the liquid/gas surface.

We found that the formation of large, interconnected NWNs on liquid surfaces was facilitated by the generation of gas bubbles during the reaction, which rupture at the solution surface through a mechanism reminiscent of exocytosis (Figure 2). Specifically, upon the addition of NaBH_4_ to the solution, a substantial number of gas bubbles were rapidly generated as a result of the following reaction: NaBH_4_ + 2H_2_O → NaBO_2_ + 4H_2_ (Figure 2a,b). The generation of abundant small bubbles dramatically increased the surface area of the gas–liquid interface to favor the formation of 2-D interfacial assemblies. NWN sheets were formed at the gas/liquid interfaces of these bubbles, as depicted in Figure 2b,f(2). When a bubble emerged to the top of the solution, it ruptured, and the NWN sheet resting on the bubble surface was released to the solution surface like exocytosis (Figure 2c,f(3)). Circular vestiges remained in the final NWN sheet, indicating that the NWN sheet originated from the bubbles and fused into a cohesive piece at the liquid surface (Figure 2d,f(4)). Artificially breaking the fused NWN sheet revealed the integrity and continuity of the interconnected network as a cohesive whole, as demonstrated by the neat fracture edges that did not follow the circular vestiges left by the bubbles (Figure 2e, Figure S4). Furthermore, we found that a flat liquid surface was crucial for the uniform distribution of bubbles, which, in turn, enhanced their ability to efficiently rupture and release the NWNs from the bubble to the surface (Figure S5).

**Figure 3:**
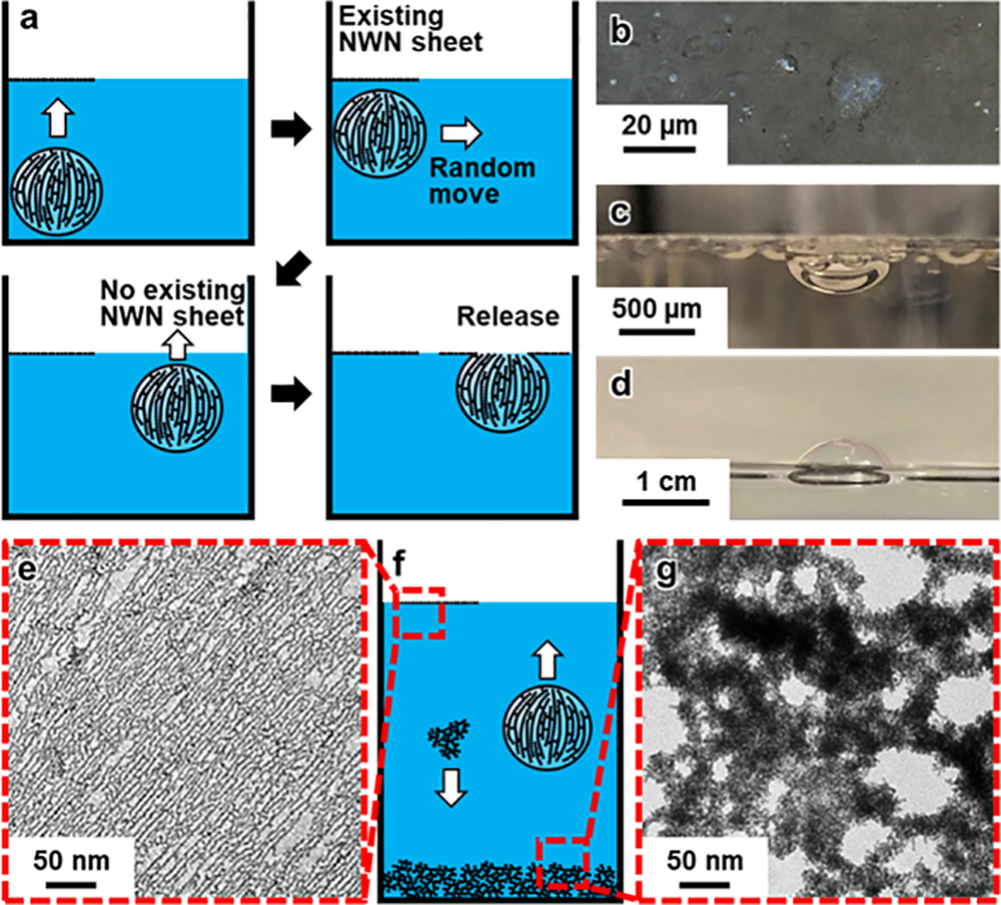
Bubble-mediated self-limiting monolayer assembly of NWN sheet at the liquid surface. (a) Schematics showing bubble rupture only occurring at surfaces without existing NWN sheet. (b,c) Bubbles underneath the liquid surface suppressed by the existing NWN sheet layer, preventing them from rising and rupturing. (b) Dark-field optical microscopy (OM) top view image. (c) Bright-field side view image. (d) A bubble without NWN sheet suppression floating above the liquid surface. (e,g) Irregular nanowire agglomeration formed outside the gas/liquid interface were not carried by the bubbles and precipitated.

The bubble-mediated assembly mechanism guaranteed a self-limiting monolayer assembly at the liquid surface. It was observed that a bubble could not rupture and release NWN beneath another existing NWN sheet on the solution surface. In such cases, the pre-existing NWN sheet acted as a suppressor that prevented the bubbles from contacting the air and ultimately hindering its rupture (Figure 3a–d).^47^ This prevented the formation of multiple layers of the NWN sheet and promoted the development of a uniform monolayer structure. We also observed that although irregular Pt nanowire aggregates did form in the solution, they precipitated to the bottom of the solution under gravity (Figure 3e–g). At a higher concentration of NaBH_4_ (8 mM), the Pt nanocrystal formation and assembly rates were accelerated, resulting in more irregular aggregations due to the lack of time to assemble on the bubble/solution interface. While the majority of the agglomeration precipitated to the bottom, some isolated small aggregates might appear in the NWN sheet at the liquid surface, possibly due to Brownian motion (Figure S6).

### Superior Electrocatalytic Performance Rising from the Anisotropic Hierarchical Structure

**Figure 4:**
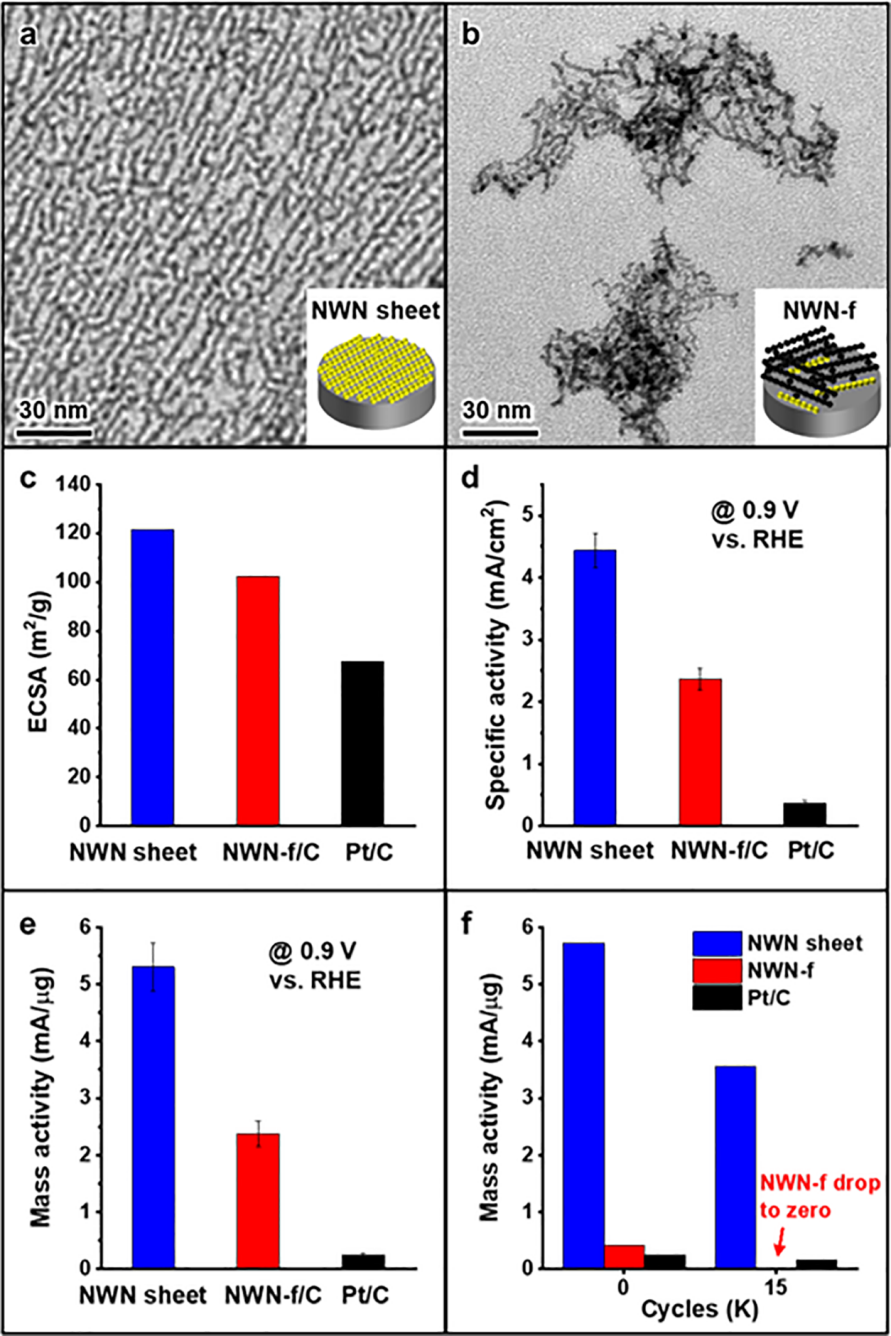
Hierarchical anisotropic assembly resulted in a superior ORR performance of NWN sheet. (a) A TEM image and a schematic inlet showing NWN sheet and its contact to the electrode. (b) A TEM image and a schematic inlet showing NWN-fragment (NWN-f) and its contact to the electrode. The sites having contact with the electrode were labeled as yellow in (a) and (b). (c) Electrochemical surface areas of different nanostructures. (d) Specific activities and (e) mass activities of different nanostructures. (f) Accelerated durability tests after 15,000 cycles.

Pt-based nanomaterials have exhibited exceptional performance in electrocatalytic applications,^48−54^ such as oxygen reduction reaction (ORR)^32,38,55−58^ and hydrogen evolution reactions (HER).^59,60^ However, challenges persist arising from sluggish oxygen reduction kinetics and high catalyst costs.^33,61^ The Pt NWN sheet, as a hierarchical anisotropic assembly, offered many benefits as an effective electrocatalyst. First, the nanowires contained abundant grain boundaries served as active catalytic sites.^36,62,63^ Second, unlike long-chain polymers, the peptides in NWN sheets were short and readily removable.^64,65^ Third, the NWN sheets could be easily transferred layer by layer onto a rotation-disk electrode, thereby forming a stacking structure (Figure S7). This enabled the use of plasma to generate a cleaner surface in NWN sheets apart from annealing for powders.^66−68^ Lastly, the structured nanowires featured uniform gaps, ensuring total catalytic site exposure and numerous contact points with neighboring surfaces (Figure 4a,b). These features resulted in the Pt NWN sheets exhibiting superior activity and stability in reactions such as the ORR and HER.

Upon synthesis, the NWN sheet was transferred onto the surface of a rotating disk electrode made of glassy carbon and subsequently underwent H_2_/O_2_ plasma treatment. The electrocatalytic performance of the Pt NWN sheet was assessed in comparison to annealed Pt NWN-fragment (NWN-f) supported on Vulcan XC-72 carbon (denoted as NWN-f/C, Figure S8) and the state-of-the-art commercial Johnson Matthey (JM) Pt/C catalyst. (See details in the Experimental Methods section.) Cyclic voltammograms and polarization curves for the ORR were recorded for NWN sheet, NWN-f/C, and Pt/C (Figure S9a,b). The onset potential of the Pt NWN-f/C and NWN sheet was approximately 0.99 and 0.98 V, respectively [all potentials reported herein are with respect to the reversible hydrogen electrode (RHE)]. Both values exceeded the onset potential of Pt/C, which was approximately 0.96 V, indicating greater ORR activities resulting from the abundant grain boundaries present in the NWN sheet and NWN-f/C.^36,62^ Electrochemical surface areas (ECSAs) in Figure 4c were determined by measuring the charge associated with the underpotential deposition of hydrogen in the cyclic voltammograms (Figure S9a). (See details in the Experimental Methods section.) Compared to the 67.57 m^2^/g_Pt_ ECSA of commercial Pt/C with a diameter of approximately 4–5 nm, the A NWN sheet was a 2-D monolayer that consists of 1-D nanowires with a thickness of ∼1.9 nm, demonstrating the highest ECSA of 121.68 m^2^/g_Pt_. In comparison, NWN-f/C showed an impressive ECSA of 102.45 m^2^/g_Pt_. The lower ECSA observed in the NWN-f/C implied that some surface sites in the material were inaccessible, which may be attributed to incomplete surface cleaning and significant blockage caused by the overlapping of NWN fragments on the carbon. As depicted in Figure 4d and Figure 4e, at 0.9 V, the specific activity (4.44 mA/cm^2^) and mass activity (5.30 mA/μg_Pt_) of the Pt NWN sheet were, respectively, 12.0 and 21.2 times higher than those of commercial Pt/C (specific activity 0.37 mA/cm^2^, mass activity 0.25 mA/μg_Pt_). Furthermore, both the specific and mass activities of the NWN sheet surpassed those of NWN-f/C (specific activity 2.36 mA/cm^2^, mass activity 2.38 mA/μg_Pt_), which could be attributed to the clean surface and the superior contact of the NWN sheet with the electrode (Figure 4a,b). Impressively, the ORR activity of the Pt NWN sheet even outperformed that of some state-of-the-art Pt alloys (Table S1).

In addition, the accelerated durability test (ADT) results revealed that even after 15,000 cycles, the NWN sheet retained a mass activity of 3.56 mA/μg_Pt_ at 0.9 V, which was still 21.3 times higher than that of Pt/C (mass activity of 0.16 mA/μg_Pt_ at 0.9 V after 15,000 cycles) (Figure 4f). It is well established that carbon-supported nanocatalysts exhibit enhanced stability due to strong metal–support interactions.^69−71^ The NWN sheet, however, was support-free, yet its mass activity drop in percentage after 15,000 cycles was comparable to Pt/C, with approximately 65% activity retained. The excellent stability can be attributed to the abundant interconnections among neighboring NWN sheet layers and the NWN sheet/electrode interface. In contrast, NWN-f, which was drop-coated onto an electrode, exhibited a dramatic decrease in stability. After 15,000 ADT cycles, NWN-f failed to achieve the limiting diffusion current, and the ORR activity at 0.9 V dropped to near zero (Figure 4f, Figure S9e). In addition to the ORR, the Pt NWN sheet also exhibited superior HER performance in 0.1 M HClO_4_ (Figure S10a). (See details in the Experimental Methods section.) The overpotential of the NWN sheet at 10 mA/cm^2^ was 6.0 mV, which was 5.2 times lower than that of commercial Pt/C (overpotential of 31.2 mV at 10 mA/cm^2^) (Figure S10b). This performance ranked among the lowest overpotentials for state-of-the-art Pt-based HER catalysts (Table S2).

## Conclusions

In summary, we have reported a bubble mediated hierarchically assembly process to achieve large-scale ultrathin Pt NWN sheets with high-density grain boundaries. This process involves the sequential assembly of 0-D ultrasmall nanocrystals into 1-D ultrathin nanowires, followed by 2-D networks and then to 3-D layered stacks. The 2-D assembly process is assisted by bubbles, and the ultrahigh anisotropy (sheet length/sheet thickness) is driven by gas/liquid surface tension. The resulting NWN sheet exhibited superior performance in various electrocatalytic reactions such as the ORR and HER owing to its clean surface and hierarchical anisotropic assembly. Our results demonstrate the potential of utilizing surface tension to engineer hierarchical anisotropic structures as a promising strategy for developing next-generation electrocatalysts.

## Experimental Methods

### Peptide Synthesis and Characterization

The peptides were synthesized via a fmoc solid-phase peptide synthesis procedure, utilizing a CS 336X synthesizer (CS Bio). (See details of chemicals in the Supporting Information.) To minimize interfering electrostatic interactions, the N- and C-terminals were acylated and amidated, respectively. Briefly, 0.5 g of fmoc-rink amide MBHA resin was deprotected with piperidine/DMF (1:4) before coupling amino acids in reverse order using fmoc-protected amino acid/HBTU/DIEA/DMF (2.5:2.25:5:90.25). Subsequently, the N-terminus was acylated with (Ac)_2_O/DIEA/DMF (5:5:90). The peptides were ultimately cleaved from the resin using phenol/H_2_O/TIPS/TFA (5:5:2:88), and the cleavage solution was precipitated into diethyl ether. The peptides were washed with diethyl ether five times and dried under a vacuum, followed by dissolution in water and subsequent freeze-drying.

### Synthesis and Characterization of the NWN Sheet

To synthesize Pt NWN sheets, a mixture of 1 mL of H_2_PtCl_6_ (10 mM), 0.6 mL of BP7A peptide (2 mg/mL), and 7.7 mL of H_2_O was prepared in a glass vial. A freshly prepared solution of NaBH_4_ (80 mM) and ascorbic acid (100 mM) was added to the mixture. The injection of 0.5 mL of ascorbic acid was followed by 0.2 mL of NaBH_4_ while stirring at 1500 rpm, which was then reduced to 300 rpm after a few seconds. To keep a flat liquid surface, the water amount was adjusted to maintain the reaction volume at 10 mL. Most bubbles formed within the initial 3–5 min, followed by a rapid decrease. To ensure that the films originating from individual bubbles had sufficient time to merge into a single piece, the final product was collected/transferred after 30 min.

For TEM characterization, the floating sheet was transferred onto a carbon-coated copper TEM grid and rinsed with water before air-drying. TEM images were captured on either an FEI T12 or JEOL JEM120-EX transmission electron microscope. HRTEM was carried out by using an FEI TITAN scanning/transmission electron microscope at 300 kV. FFT diffraction patterns were generated by using ImageJ.

For SEM characterization, the NWN sheet was transferred onto a SiO_2_-coated Si wafer and dipped in water before being subjected to H_2_/O_2_ plasma cleaning for 10 min intervals using the Gatan plasma system SOLARUS. SEM images were captured using a Nova Nano 230 scanning electron microscope.

### Analysis of Surface Tension and Contact Angles

Surface tension measurement was conducted on a Kruss K100 force tensiometer using the Wilhelmy plate method. To ensure complete coverage, we transferred five layers of NWN sheets onto a silica wafer before measuring the static and dynamic contact angles. The static contact angle was measured using drop shape analysis on sessile drops, while the dynamic contact angle was measured through shape analysis of a drop that moves over the inclined surface of the NWN-sheet-coated wafer.

### Synthesis and Characterization of NWN-f

To synthesize NWN-f, the procedure was similar to that of NWN sheets, except that the stirring speed was fixed at 2000 rpm. This vigorous stirring ensures the effective breakup of the NWN sheets during their formation at the gas/liquid interface of bubbles, resulting in the production of NWN-f. The synthesis was completed in 30 min. To prepare the TEM grids, samples of the reaction mixture were extracted and transferred onto carbon-coated copper TEM grids using pipets. These grids were then washed with water and air-dried at room temperature.

### NWN-f Dispersion and Annealing on Carbon

To prepare NWN-f/C, NWN-f was mixed with Vulcan XC72R carbon and sonicated overnight. The resulting mixture was then washed with 0.1 M HClO_4_ and water and annealed in air at 150 °C for 15 min. The Pt loading was maintained at approximately 20 wt %.

### NWN Sheets Transfer onto Electrode and Plasma Cleaning

To transfer the as-synthesized floating NWN sheets onto a glassy carbon rotating disk electrode, the electrode was simply dipped onto the liquid surface, where the NWN sheets were floating. The transferred electrode was then washed by being dipped in clean water several times and allowed to dry. To achieve thorough cleaning, the electrode was treated with H_2_/O_2_ plasma in the Gatan Plasma System SOLARUS for 10 min, with intervals in between. For electrochemical tests, the transfer process was repeated, followed by another cleaning, resulting in a final electrode with two layers of NWN sheets.

### Electrochemical Measurements of the Oxygen Reduction Reaction (ORR)

To determine the Pt loading, inductively coupled plasma-atomic emission spectroscopy (ICP-AES) was used. The average and area normalization of multiple large-area transfers were employed to calculate the Pt loading of NWN sheets on the electrode, which was estimated as 0.157 μg. For the deposition of NWN-f/C, Pt/C, and NWN-f, ethanol dispersions of the samples were deposited on glassy carbon rotating disk electrodes to obtain the working electrodes. The Pt loading amount is 2 μg for Pt NWN-f/C and commercial Pt/C and 5 μg for Pt NWN-f. The reference electrode used was a silver chloride electrode, and all potentials were measured relative to the reversible hydrogen electrode (RHE). Cyclic voltammograms at a sweep rate of 50 mV/s were conducted in a N_2_ saturated 0.1 M perchloric acid solution until the curves between the 2 measurements were close to identical. The polarization curves were recorded at a sweep rate of 20 mV/s in an oxygen-saturated 0.1 M perchloric acid solution, with the working electrode rotating at 1600 rpm. The current was normalized with the geometric area of the electrode (0.196 cm^2^) to get the current density, and all electrode potentials were recorded with respect to RHE after IR correction.^72^ An accelerated durability test (ADT) was conducted by cycling the potential between 0.6 and 1.0 V (vs RHE) in oxygen-saturated solutions at a scan rate of 100 mV/s. Electrochemical surface area (ECSA) was estimated by measuring the charge associated with the underpotential deposition of hydrogen (*Q*_*H*_) and assuming 210 μC/cm^2^ for the adsorption of a monolayer of hydrogen on the surface. The specific ECSA was then calculated using the formula: . For the ORR at an RDE, the Koutecky–Levich equation, , was used to calculate the kinetic current (*i*_*k*_) and diffusion-limiting current (*i*_*d*_) based on the experimentally measured current (*i*). The specific activity at 0.9 V was obtained by normalizing the kinetic current at 0.9 V to that of the ECSA.

### Electrochemical Measurements of the Hydrogen Evolution Reaction (HER)

To load both NWN sheets and commercial Pt/C, the same method as that used in the ORR measurements was employed, with a Pt loading amount of 0.157 μg for both catalysts. The working electrode consisted of a glassy carbon rotating disk electrode coated with the appropriate catalyst, while the reference electrode was a silver chloride electrode. A graphite rod was utilized as the counter electrode, and the electrolyte was 0.1 M HClO_4_ that had been saturated by N_2_. The HER was evaluated at a sweep rate of 5 mV/s, and all electrode potentials were recorded relative to the RHE following the IR correction.

## Supporting Information Available

Chemicals; dynamic contact angle analysis; OM, SEM, and TEM images; BP7A peptide influence on NWN sheet morphology; surface tension measurements; bubble distribution; transfer and stacking process; CV, ORR polarization curve, and ADT results; hierarchical anisotropic assembly gives superior HER performance; tables of ORR specific and mass activities and of HER overpotentials (PDF)
