# Supplementary material for: Bubble-Mediated Large-Scale Hierarchical Assembly of Ultrathin Pt Nanowire Network Monolayer at Gas/Liquid Interfaces
Source: ACS Nano. 2023 Jul 6;17(14):14152–60. doi: 10.1021/acsnano.3c04771 (PMC10373521; doi:10.1021/acsnano.3c04771)
Supplement: Supplementary file 1 — nn3c04771_si_001.pdf [file nn3c04771_si_001.pdf]

Supplementary Information

Bubble-Mediated Large-Scale Hierarchical Assembly of Ultrathin Pt Nanowire Network Monolayer at Gas/Liquid Interfaces

Enbo Zhu,[†,#] Yang Liu,,[†,#] Jin Huang,[†] Ao Zhang,[†] Bosi Peng,[Δ] Zeyan Liu,[†] Haotian Liu,[†] Jiaji Yu,[‡] Yan-Ruide Li,[‡] Lili Yang,[‡] Xiangfeng Duan,[Δ] and Yu Huang[,†,ǁ]*

[†]Department of Materials Science and Engineering, [Δ]Department of Chemistry and Biochemistry, [‡]Department of Microbiology, Immunology & Molecular Genetics, [ǁ]California NanoSystems Institute, University of California, Los Angeles, CA 90095, USA.

[#] Enbo Zhu and Yang Liu contribute equally

**To whom correspondence should be addressed. E-mail:* yhuang@seas.ucla.edu

CONTENTS

This PDF file includes: Chemicals; Dynamic contact angle analysis; Figures S1 to S8; Table S1; References

1. Chemicals

Fmoc-rink amide MBHA resin (AnaSpec), fmoc-protected amino acids (AnaSpec), piperidine (Sigma-Aldrich), o-benzotriazole-N,N,N',N'-tetramethyl-uronium-hexafluoro-phosphate (HBTU, AnaSpec), N,N-diisopropylethylamine (DIEA, Sigma-Aldrich), acetic anhydride ($(AC)_2O$, Fisher Scientific), trifluoroacetic acid (TFA, Sigma-Aldrich), triisopropylsilane (TIPS, Sigma-Aldrich), phenol (Sigma-Aldrich), dimethylformamide (DMF, EMD), dichloromethane (DCM, Fisher Scientific), diethyl ether (Fisher Scientific), chloroplatinic acid hydrate ($H_2Pt(IV)Cl_6.xH_2O$, 99.9+ % trace metals basis, Sigma-Aldrich), sodium borohydride ($NaBH_4$, Sigma-Aldrich), ascorbic acid ($C_6H_8O_6$, Sigma-Aldrich). All chemicals were applied as received without further purification. Commercial Pt/C catalysts (HiSPECTM3000, 20 wt.% Pt on Vulcan XC72R carbon) purchased from Johnson Matthey were used for comparison.

2. Analyzing Dynamic Contact Angle of a Nanowire Pinned at the Gas-Liquid Interface

The Main Manuscript Derives Energy Reduction by Surface Tension as:

$$\Delta G = 2LR\theta\gamma_{LG}cos\theta - 2LRsin\theta\gamma_{LG} = 2LR\gamma_{LG}(\theta cos\theta - sin\theta)$$

where R = 0.95 nm; γ_{LG} = 68.64 mN/m. Based on Dynamic Contact Angles, with advancing angle $\theta_a = 0.24\pi$, and receding angle $\theta_r = 0.11\pi$, contact angle range is $0.11\pi < \theta < 0.24\pi$. Thus:

$$-1.76 \times 10^{-20}L\ (J/nm) < \Delta G < -1.77 \times 10^{-21}L\ (J/nm)$$

At room temperature (20 °C), $kT = -4.04 \times 10^{-21}J$. For $\Delta G < -10kT$, 2.30 nm < L < 22.82 nm, and 2 < n < 12 nanocrystals of 1.9 nm Size are Required. For $\Delta G < -100kT$, 23.0 nm < L < 228.2 nm, and 13 < n < 120 nanocrystals of 1.9 nm size are required.

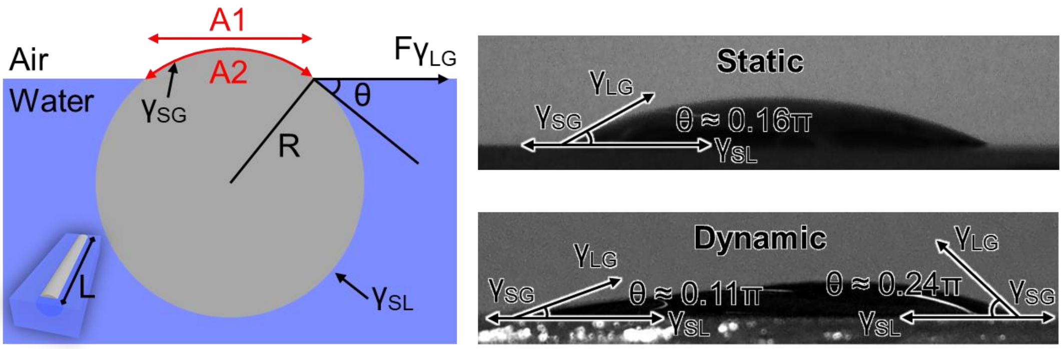

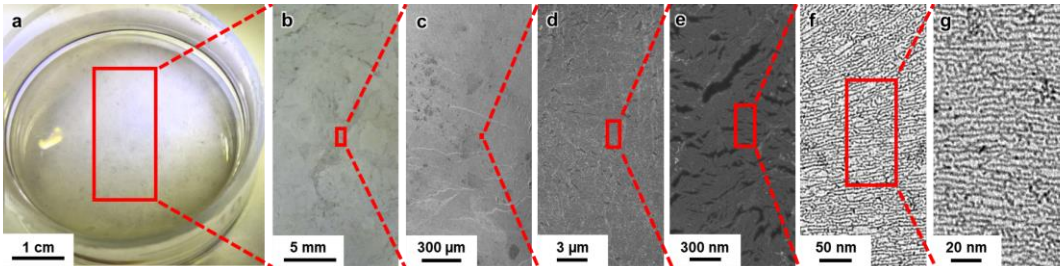

Figure S1. Large-scale nanowire network (NWN) sheets formed on the liquid surface. (a-g) Sequential magnification of representative images captured at different scales for a comprehensive understanding. (a-b) Bright-field optical microscopy (OM) images; (c-e) Scanning electron microscopy (SEM) images; (f-g) Transmission electron microscopy (TEM) images.

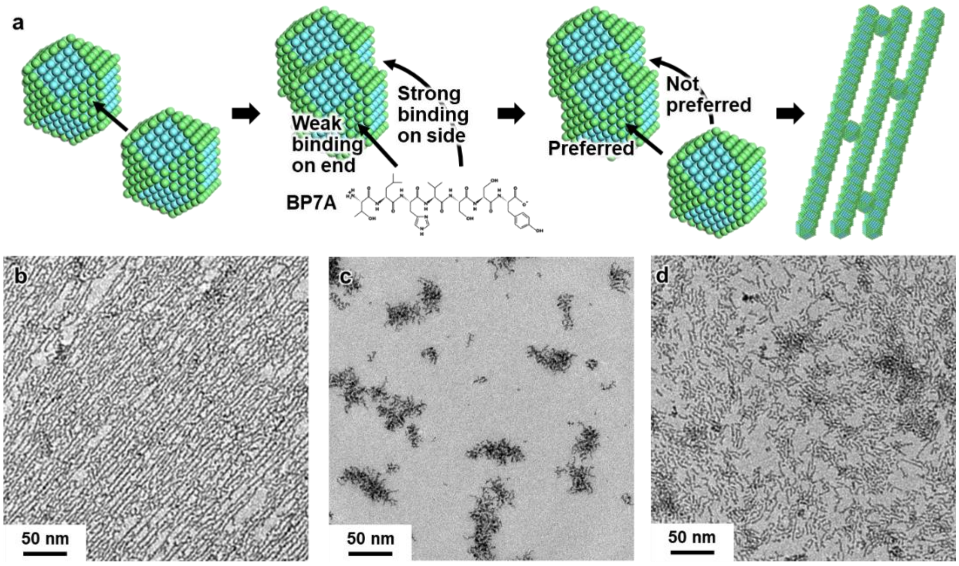

Figure S2. Influence of BP7A peptides on the morphology of NWN sheets. (a) binding mechanism of BP7A to grain boundaries and the resulting morphology of the NWN structure. (b-d) The effect of varying BP7A concentrations on the morphology of the NWN sheets. (b) 20 µg/mL. (c) 120 µg/mL. (d) 400 µg/mL.

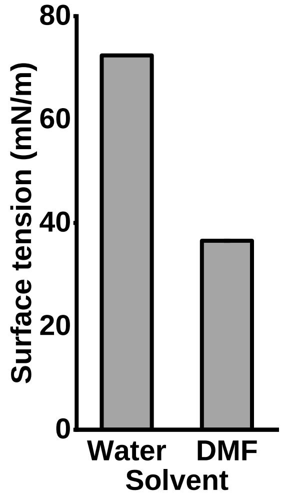

Figure S3. Surface tension measurements of different solvents at a temperature of 20 °C. Water demonstrates a markedly higher surface tension compared to DMF.

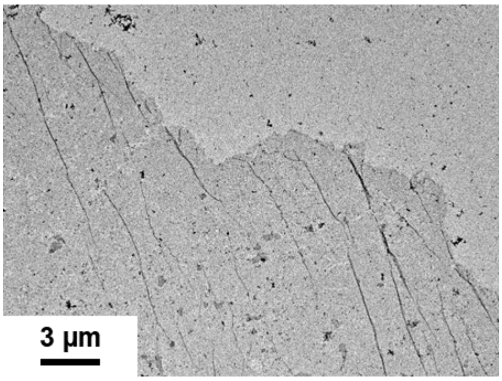

Figure S4. TEM image demonstrating the fracture edge of a NWN sheet. The sharp edges in the image suggest that the nanowire networks are well connected, forming a cohesive and continuous sheet. The lack of a discernible pattern in the breakage further highlights the mechanical homogeneity of the final NWN sheet.

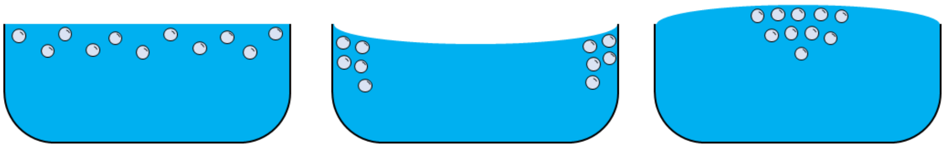

Figure S5. Distribution of bubbles under different surface curvatures. A flat curvature resulted in a well-distributed bubble pattern, while a positive or negative curvature caused bubble aggregations.

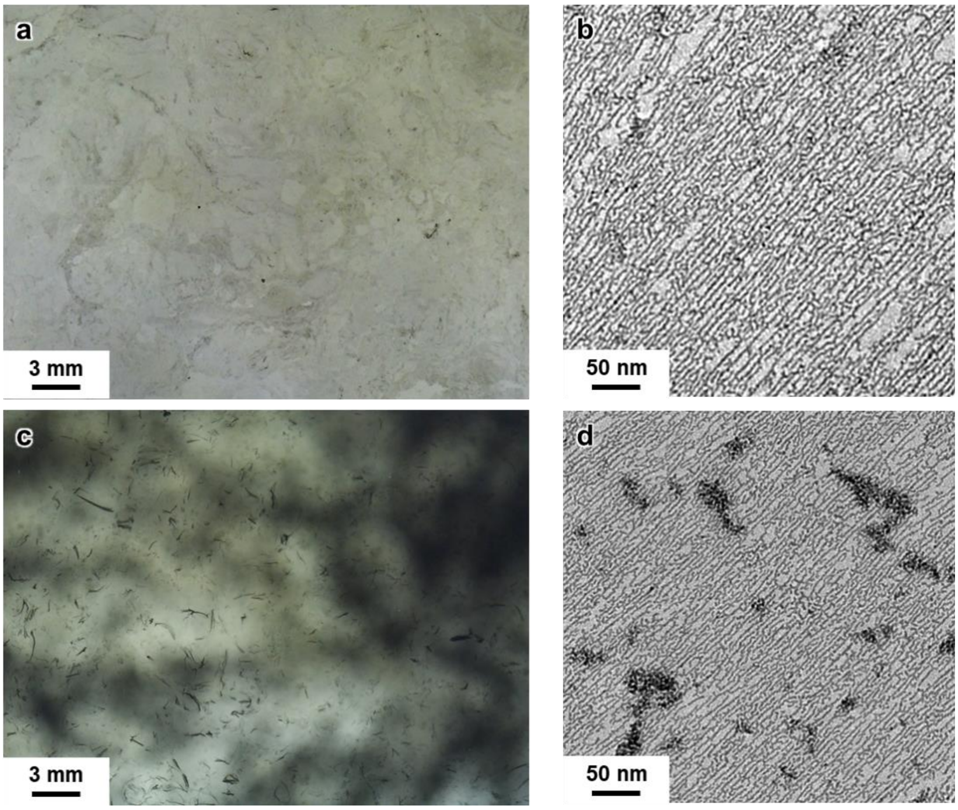

Figure S6. Effect of $NaBH_4$ concentration on the synthesis of NWN sheets. (a-b) NWN sheets prepared using $NaBH_4$ concentrations of 1.6 mM. (c-d) NWN sheets prepared using $NaBH_4$ concentrations of 8 mM. TEM images in (b) and (d) show the resulting NWN sheets floating on the surface of the liquid.

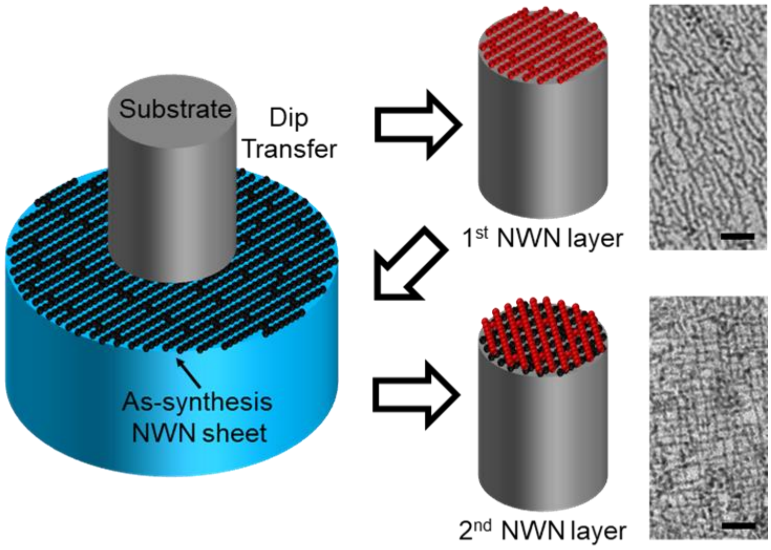

Figure S7. Schematics showing layer-to-layer transfer and stacking process. The most recent transferred layer is labelled as red. Scale bars: 20 nm.

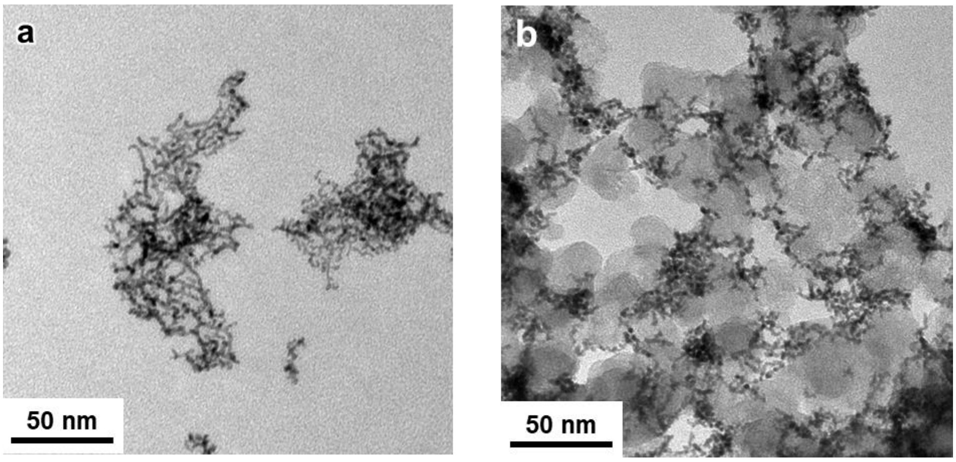

Figure S8. TEM images of NWN-fragment (NWN-f) before and after loading onto Vulcan XC-72 carbon. (a) The dispersed NWN-f formed tangled network of interconnected nanowires with confined size. (b) NWN-f dispersed well on the carbon substrate.

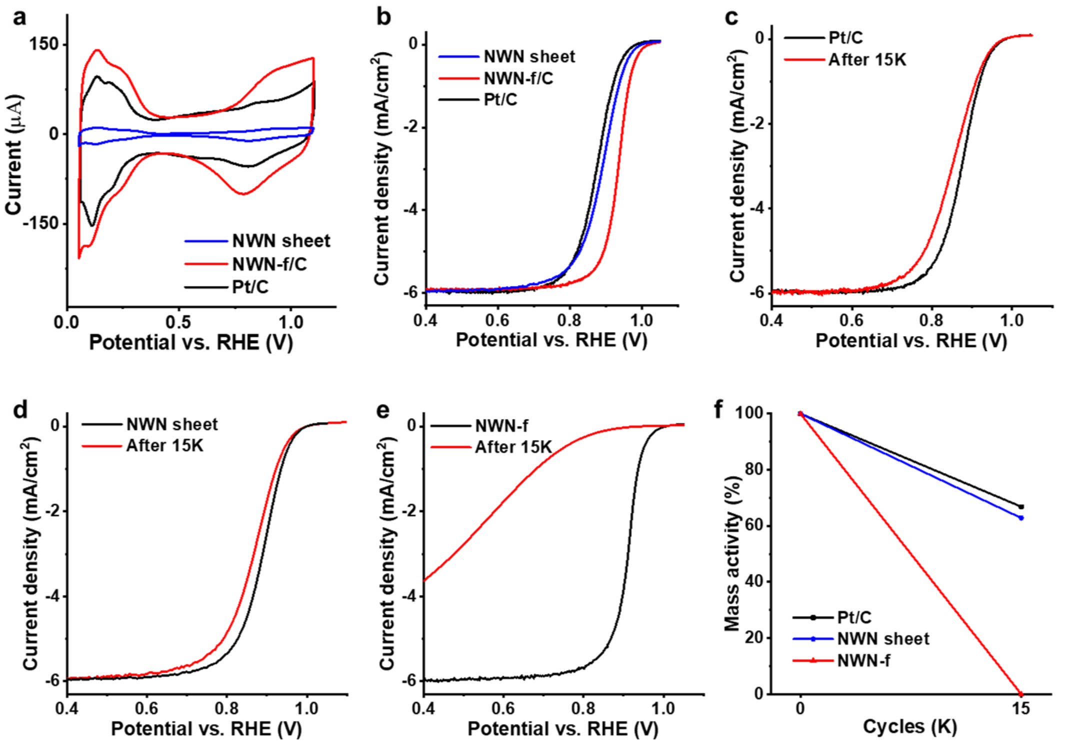

Figure S9. Cyclic voltammogram (CV), ORR polarization curve, and accelerated durability test (ADT) on different samples. (a) The CVs and (b) ORR polarization curves of NWN sheet, NWN-f/C, and Pt/C. (c-e) The ORR polarization curves of (c) Pt/C, (d) NWN sheet, and (e) NWN-f before and after 15,000 cycles. (f) Percentage drop in mass activities during the ADT.

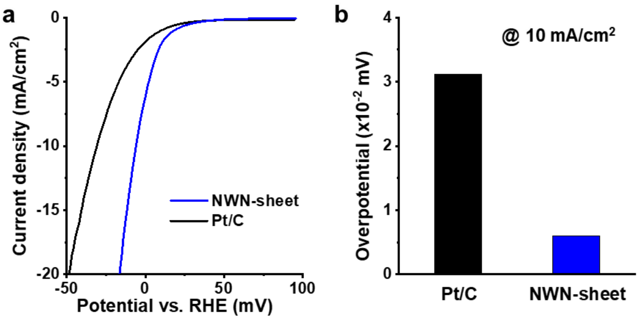

Figure S10. Hierarchical anisotropic assembly resulted in superior HER performance of NWN sheet. (a) HER polarization curves of different nanostructures. (b) HER overpotential of different nanostructures at 10 mA/cm^2.

Table S1. ORR specific activities (SA) and mass activities (MA) of state-of-art Pt-based nano-catalysts from recently published works. Electrolyte: 0.1 M $HClO_4$. Current density @ 0.9 V vs. RHE.

Catalyst	SA (mA cm^{-2})	MA (A mg_{metal}^{-1})
**Pt nanowire network sheet (this work)**	**4.44**	**5.30**
Pt NWN-f/C (this work)	2.36	2.38
Commercial Pt/C (this work)	0.37	0.25
Pt Nanoparticles[1]	0.87	0.99
Jagged-Pt nanowires[2]	11.5	13.6
Star-shaped $AuPt_{1.03}$/C[3]	1.09	0.47
Pd@Pt Nanowire[4]	0.98	0.516
Pt_3Co Nanoparticles/C[5]	1.00	0.52
Sub-Pt_3Co-MC[6]	1.74	0.84
Pt_3Co@Pt-SAC[7]	3.5	~1.27
Silver Templated Pt Nanoplates[8]	5.3	1.46
PtPb Nanoplates/C[9]	7.8	2.086
Pt_xY Nanoparticles[10]	~13	~2.84
$Pt_{2.5}Ni$ Octahedra/C[11]	N/A	2.95
Pd-doped Pt nanoplate/C[12]	6.01	3.62
Pt_3Ni/C Nanoframes[13]	N/A	5.18
Mo-PtNi Nanooctahedra/C[14]	10.3	5.37
Pt-Ni Hexoctahedra[15]	1.08	N/A
Pt_3Al/Pt/C[16]	1.23	N/A
Pd@Pt Nanoicosahedra/C[17]	1.36	N/A

Table S2. HER overpotentials of state-of-art Pt-based nano-catalysts from recently published works. Electrolyte: 0.1 M $HClO_4$. Overpotential @ 10 mA/cm^2

Catalyst	Overpotential (mV)
**Pt nanowire network sheet (this work)**	**6.0**
Commercial Pt/C (this work)	31.2
PtP2@PNC[18]	8
WO_x-PtNi@Pt DNWs[19]	5
NiS_2/PtNi[20]	15
TePtFe NTs[21]	28.1

References

(1) Garlyyev, B.; Kratzl, K.; Rück, M.; Michalička, J.; Fichtner, J.; Macak, J. M.; Kratky, T.; Günther, S.; Cokoja, M.; Bandarenka, A. S.; Gagliardi, A.; Fischer, R. A. Optimizing the Size of Platinum Nanoparticles for Enhanced Mass Activity in the Electrochemical Oxygen Reduction Reaction. *Angew. Chem. Int. Ed.* **2019**, *58* (28), 9596–9600. https://doi.org/10.1002/anie.201904492.

(2) Li, M.; Zhao, Z.; Cheng, T.; Fortunelli, A.; Chen, C.-Y.; Yu, R.; Zhang, Q.; Gu, L.; Merinov, B. V.; Lin, Z.; Zhu, E.; Yu, T.; Jia, Q.; Guo, J.; Zhang, L.; Goddard, W. A.; Huang, Y.; Duan, X. Ultrafine Jagged Platinum Nanowires Enable Ultrahigh Mass Activity for the Oxygen Reduction Reaction. *Science* **2016**, *354* (6318), 1414–1419. https://doi.org/10.1126/science.aaf9050.

(3) Bian, T.; Zhang, H.; Jiang, Y.; Jin, C.; Wu, J.; Yang, H.; Yang, D. Epitaxial Growth of Twinned Au–Pt Core–Shell Star-Shaped Decahedra as Highly Durable Electrocatalysts. *Nano Lett.* **2015**, *15* (12), 7808–7815. https://doi.org/10.1021/acs.nanolett.5b02960.

(4) Li, H.-H.; Ma, S.-Y.; Fu, Q.-Q.; Liu, X.-J.; Wu, L.; Yu, S.-H. Scalable Bromide-Triggered Synthesis of Pd@Pt Core–Shell Ultrathin Nanowires with Enhanced Electrocatalytic Performance toward Oxygen Reduction Reaction. *J. Am. Chem. Soc.* **2015**, *137* (24), 7862–7868. https://doi.org/10.1021/jacs.5b03877.

(5) Wang, D.; Xin, H. L.; Hovden, R.; Wang, H.; Yu, Y.; Muller, D. A.; DiSalvo, F. J.; Abruña, H. D. Structurally Ordered Intermetallic Platinum–Cobalt Core–Shell Nanoparticles with Enhanced Activity and Stability as Oxygen Reduction Electrocatalysts. *Nat. Mater.* **2013**, *12* (1), 81–87. https://doi.org/10.1038/nmat3458.

(6) Cheng, H.; Gui, R.; Yu, H.; Wang, C.; Liu, S.; Liu, H.; Zhou, T.; Zhang, N.; Zheng, X.; Chu, W.; Lin, Y.; Wu, H.; Wu, C.; Xie, Y. Subsize Pt-Based Intermetallic Compound Enables Long-Term Cyclic Mass Activity for Fuel-Cell Oxygen Reduction. *Proc. Natl. Acad. Sci.* **2021**, *118* (35), e2104026118. https://doi.org/10.1073/pnas.2104026118.

(7) Liu, B.; Feng, R.; Busch, M.; Wang, S.; Wu, H.; Liu, P.; Gu, J.; Bahadoran, A.; Matsumura, D.; Tsuji, T.; Zhang, D.; Song, F.; Liu, Q. Synergistic Hybrid Electrocatalysts of Platinum Alloy and Single-Atom Platinum for an Efficient and Durable Oxygen Reduction Reaction. *ACS Nano* **2022**, *16* (9), 14121–14133. https://doi.org/10.1021/acsnano.2c04077.

(8) Liu, H.; Zhong, P.; Liu, K.; Han, L.; Zheng, H.; Yin, Y.; Gao, C. Synthesis of Ultrathin Platinum Nanoplates for Enhanced Oxygen Reduction Activity. *Chem. Sci.* **2018**, *9* (2), 398–404. https://doi.org/10.1039/C7SC02997G.

(9) Bu, L.; Zhang, N.; Guo, S.; Zhang, X.; Li, J.; Yao, J.; Wu, T.; Lu, G.; Ma, J.-Y.; Su, D.; Huang, X. Biaxially Strained PtPb/Pt Core/Shell Nanoplate Boosts Oxygen Reduction Catalysis. *Science* **2016**, *354* (6318), 1410–1414. https://doi.org/10.1126/science.aah6133.

(10) Hernandez-Fernandez, P.; Masini, F.; McCarthy, D. N.; Strebel, C. E.; Friebel, D.; Deiana, D.; Malacrida, P.; Nierhoff, A.; Bodin, A.; Wise, A. M.; Nielsen, J. H.;

Hansen, T. W.; Nilsson, A.; Stephens, I. E. L.; Chorkendorff, I. Mass-Selected Nanoparticles of PtxY as Model Catalysts for Oxygen Electroreduction. *Nat. Chem.* **2014**, *6* (8), 732–738. https://doi.org/10.1038/nchem.2001.
(11) Choi, S.-I.; Xie, S.; Shao, M.; Odell, J. H.; Lu, N.; Peng, H.-C.; Protsailo, L.; Guerrero, S.; Park, J.; Xia, X.; Wang, J.; Kim, M. J.; Xia, Y. Synthesis and Characterization of 9 Nm Pt-Ni Octahedra with a Record High Activity of 3.3 A/Mg(Pt) for the Oxygen Reduction Reaction. *Nano Lett.* **2013**, *13* (7), 3420–3425. https://doi.org/10.1021/nl401881z.
(12) Zhu, E.; Yan, X.; Wang, S.; Xu, M.; Wang, C.; Liu, H.; Huang, J.; Xue, W.; Cai, J.; Heinz, H.; Li, Y.; Huang, Y. Peptide-Assisted 2-D Assembly toward Free-Floating Ultrathin Platinum Nanoplates as Effective Electrocatalysts. *Nano Lett.* **2019**, *19* (6), 3730–3736. https://doi.org/10.1021/acs.nanolett.9b00867.
(13) Chen, C.; Kang, Y.; Huo, Z.; Zhu, Z.; Huang, W.; Xin, H. L.; Snyder, J. D.; Li, D.; Herron, J. A.; Mavrikakis, M.; Chi, M.; More, K. L.; Li, Y.; Markovic, N. M.; Somorjai, G. A.; Yang, P.; Stamenkovic, V. R. Highly Crystalline Multimetallic Nanoframes with Three-Dimensional Electrocatalytic Surfaces. *Science* **2014**, *343* (6177), 1339–1343. https://doi.org/10.1126/science.1249061.
(14) Huang, X.; Zhao, Z.; Cao, L.; Chen, Y.; Zhu, E.; Lin, Z.; Li, M.; Yan, A.; Zettl, A.; Wang, Y. M.; Duan, X.; Mueller, T.; Huang, Y. High-Performance Transition Metal–Doped Pt3Ni Octahedra for Oxygen Reduction Reaction. *Science* **2015**, *348* (6240), 1230–1234. https://doi.org/10.1126/science.aaa8765.
(15) Xu, X.; Zhang, X.; Sun, H.; Yang, Y.; Dai, X.; Gao, J.; Li, X.; Zhang, P.; Wang, H.-H.; Yu, N.-F.; Sun, S.-G. Synthesis of Pt-Ni Alloy Nanocrystals with High-Index Facets and Enhanced Electrocatalytic Properties. *Angew. Chem. Int. Ed Engl.* **2014**, *53* (46), 12522–12527. https://doi.org/10.1002/anie.201406497.
(16) Lang, X.-Y.; Han, G.-F.; Xiao, B.-B.; Gu, L.; Yang, Z.-Z.; Wen, Z.; Zhu, Y.-F.; Zhao, M.; Li, J.-C.; Jiang, Q. Mesostructured Intermetallic Compounds of Platinum and Non-Transition Metals for Enhanced Electrocatalysis of Oxygen Reduction Reaction. *Adv. Funct. Mater.* **2015**, *25* (2), 230–237. https://doi.org/10.1002/adfm.201401868.
(17) Wang, X.; Choi, S.-I.; Roling, L. T.; Luo, M.; Ma, C.; Zhang, L.; Chi, M.; Liu, J.; Xie, Z.; Herron, J. A.; Mavrikakis, M.; Xia, Y. Palladium–Platinum Core-Shell Icosahedra with Substantially Enhanced Activity and Durability towards Oxygen Reduction. *Nat. Commun.* **2015**, *6* (1), 7594. https://doi.org/10.1038/ncomms8594.
(18) Pu, Z.; Cheng, R.; Zhao, J.; Hu, Z.; Li, C.; Li, W.; Wang, P.; Amiinu, I. S.; Wang, Z.; Min Wang; Chen, D.; Mu, S. Anion-Modulated Platinum for High-Performance Multifunctional Electrocatalysis toward HER, HOR, and ORR. *iScience* **2020**, *23* (12), 101793. https://doi.org/10.1016/j.isci.2020.101793.
(19) Zhang, W.; Huang, B.; Wang, K.; Yang, W.; Lv, F.; Li, N.; Chao, Y.; Zhou, P.; Yang, Y.; Li, Y.; Zhou, J.; Zhang, W.; Du, Y.; Su, D.; Guo, S. WOx-Surface Decorated PtNi@Pt Dendritic Nanowires as Efficient PH-Universal Hydrogen Evolution Electrocatalysts. *Adv. Energy Mater.* **2021**, *11* (3), 2003192. https://doi.org/10.1002/aenm.202003192.
(20) Wang, G.; Huang, X.; Liao, H.-G.; Sun, S.-G. Microstrain Engineered NixS2/PtNi

Porous Nanowires for Boosting Hydrogen Evolution Activity. *Energy Fuels* **2021**, *35* (8), 6928–6934. https://doi.org/10.1021/acs.energyfuels.1c00626.

(21) Li, W.; Amiinu, I. S.; Ye, B.; Wang, Z.; Zhu, J.; Kou, Z.; Mu, S. TePtFe Nanotubes as High-Performing Bifunctional Electrocatalysts for the Oxygen Reduction Reaction and Hydrogen Evolution Reaction. *ChemSusChem* **2018**, *11* (8), 1328–1333. https://doi.org/10.1002/cssc.201702403.
